# Psoriasis and osteoporosis: a literature review

**DOI:** 10.1111/ced.15174

**Published:** 2022-05-25

**Authors:** Dohyen Wi, Anna Wilson, Fanny Satgé, Dédée F. Murrell

**Affiliations:** ^1^ Faculty of Medicine University of New South Wales Sydney NSW Australia; ^2^ Department of Dermatology St George Hospital Sydney NSW Australia; ^3^ Faculty of Medicine University of Paris VII, East Créteil, Val de Marne Paris France

## Abstract

Psoriasis is a chronic inflammatory skin disease with complex comorbidities. Recent evidence has revealed how the inflammatory nature of psoriasis affects bone mineral density and may lead to osteoporosis. This review outlines the current understanding and advances on the association between psoriasis and osteoporosis. The current literature suggests an increased risk of osteopenia and osteoporosis in patients with extensive and chronic psoriasis, compounded by other lifestyle and genetic factors. It suggests that prophylactic measures such as vitamin D supplementation and increasing weight‐bearing exercises can help, but in patients with extensive psoriasis, prolonged systemic inflammation may require long‐term management. Although there have been many short‐term RCTs on the efficacy and safety of biologics in psoriasis, clinical studies looking at the long‐term effects of biologics, such as whether they might improve bone mineral density in these patients with psoriasis are yet to be conducted.

## Graphical abstract

This review aims to determine the relationship between psoriasis and osteoporosis through an evaluation of the current literature by assessing the prevalence of osteopenia and osteoporosis in patients with psoriasis. Several case–control and cross‐sectional studies unveiled an association between psoriasis and osteoporosis, whereas other studies found no clear evidence. In the future, more robust and larger sample studies are required to address these limitations and subsequently substantiate these results.

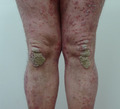

## Introduction

Over the past decade, evidence has linked psoriasis and psoriatic arthritis with cardiovascular disease and metabolic syndrome. Studies associate psoriatic arthritis with osteoporosis; however, there is limited literature on an association between psoriasis and osteoporosis. We aimed to determine if a relationship exists, through assessing the prevalence of osteopenia and osteoporosis in patients with psoriasis, and focusing on patients with severe psoriasis receiving treatment with monoclonal antibodies (biologics).

## Psoriasis

Psoriasis is an immune‐mediated chronic inflammatory skin disease affecting 2% of white populations, with its visibility causing social stigmatization, pain, discomfort and psychological distress, leading to reduced quality of life (QoL).^1^, ^2^ In psoriasis, cytokine overactivity leads to keratinocyte hyperproliferation, creating thickened inflamed plaques with silvery scale.^3^, ^4^, ^5^ Patients with visible psoriatic plaques often cover their skin and avoid sport, contributing to osteopenia.^6^ Metabolic syndrome occurs with extensive psoriasis.^7^ In the past two decades, severe psoriasis has been managed with biologics.^8^, ^9^, ^10^

## Osteopenia and osteoporosis

Osteopenia is characterized by low bone mineral density (BMD), and can progress to osteoporosis, the systemic diminished bone mass and deterioration of microarchitectural bone tissue, increasing the risk of bone fragility and fractures.^10^ Osteoporosis affects > 200 million people worldwide, approximately 20% of men and 50% of women, with a higher prevalence among postmenopausal women.^11^ Dual‐energy X‐ray absorptiometry measures BMD using T scores,^12^ measured at the lumbar or proximal femur, with scores of −2.5 to −1 SD defined as osteopenia, and scores below −2.5 SD defined as osteoporosis.^13^ Early osteoporosis diagnosis and intervention can prevent fractures.^14^ Patients are recommended adequate calcium and vitamin D intake, weight‐bearing exercises and pharmacological therapy.^14^

## Psoriasis pathophysiology and osteoporosis

**Figure 1:**
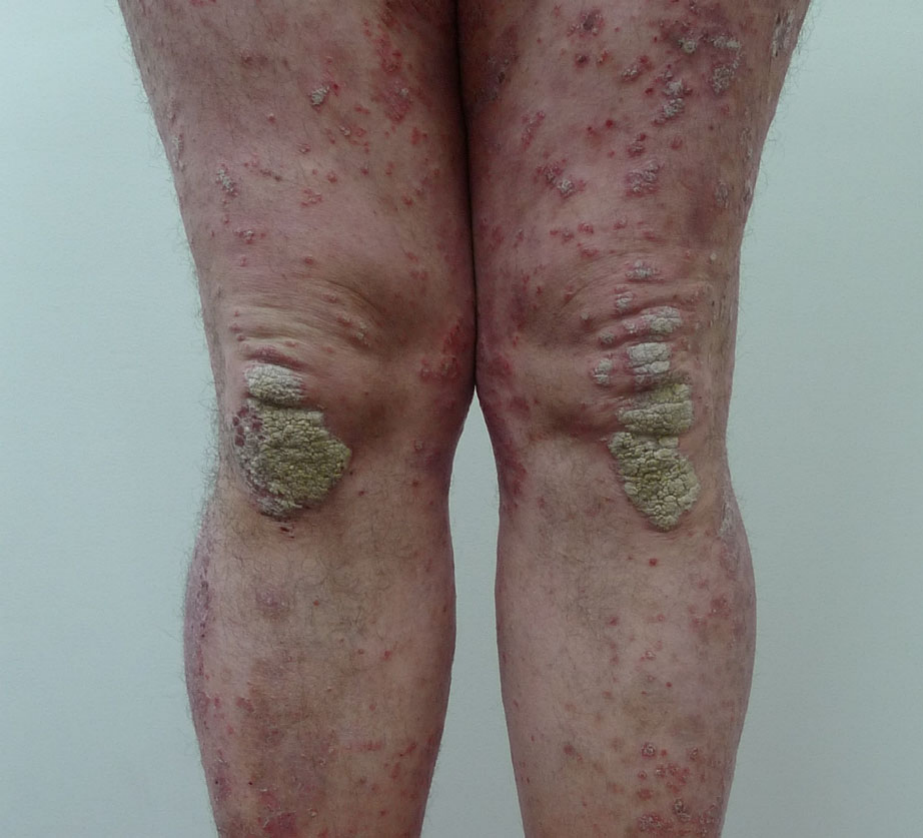
Psoriasis plaques severely impact the quality of life of patients. [Colour figure can be viewed at wileyonlinelibrary.com]

In psoriasis, systemic T‐cell activation plays a key role in the development of a T‐helper 1 type cytokine pattern with predominant secretion of interleukin (IL)‐2, IL‐6, interferon (IFN)‐γ and tumour necrosis factor (TNF)‐α. These cytokines induce abnormal proliferation and differentiation of keratinocytes, leading to psoriatic plaques^15^ (Fig. 1). IL‐6, IFN‐β and TNF‐α are involved in bone metabolism regulation and the pathogenesis of osteoporosis.^11^ TNF‐α and IL‐6 increase the production of both receptor activator of receptor activator of nuclear factor‐κB ligand and osteoprotegerin, which stimulate osteoclastogenesis.^16^

## Effects of antipsoriatic treatments on bone mineral density

Antipsoriatic drugs such as methotrexate and ciclosporin interfere with bone metabolism,^17^ and systemic corticosteroids reduce collagen genesis.^17^, ^18^, ^19^ Ultraviolet light therapy increases vitamin D and improves BMD.^14^, ^17^

## Literature search

**Figure 2:**
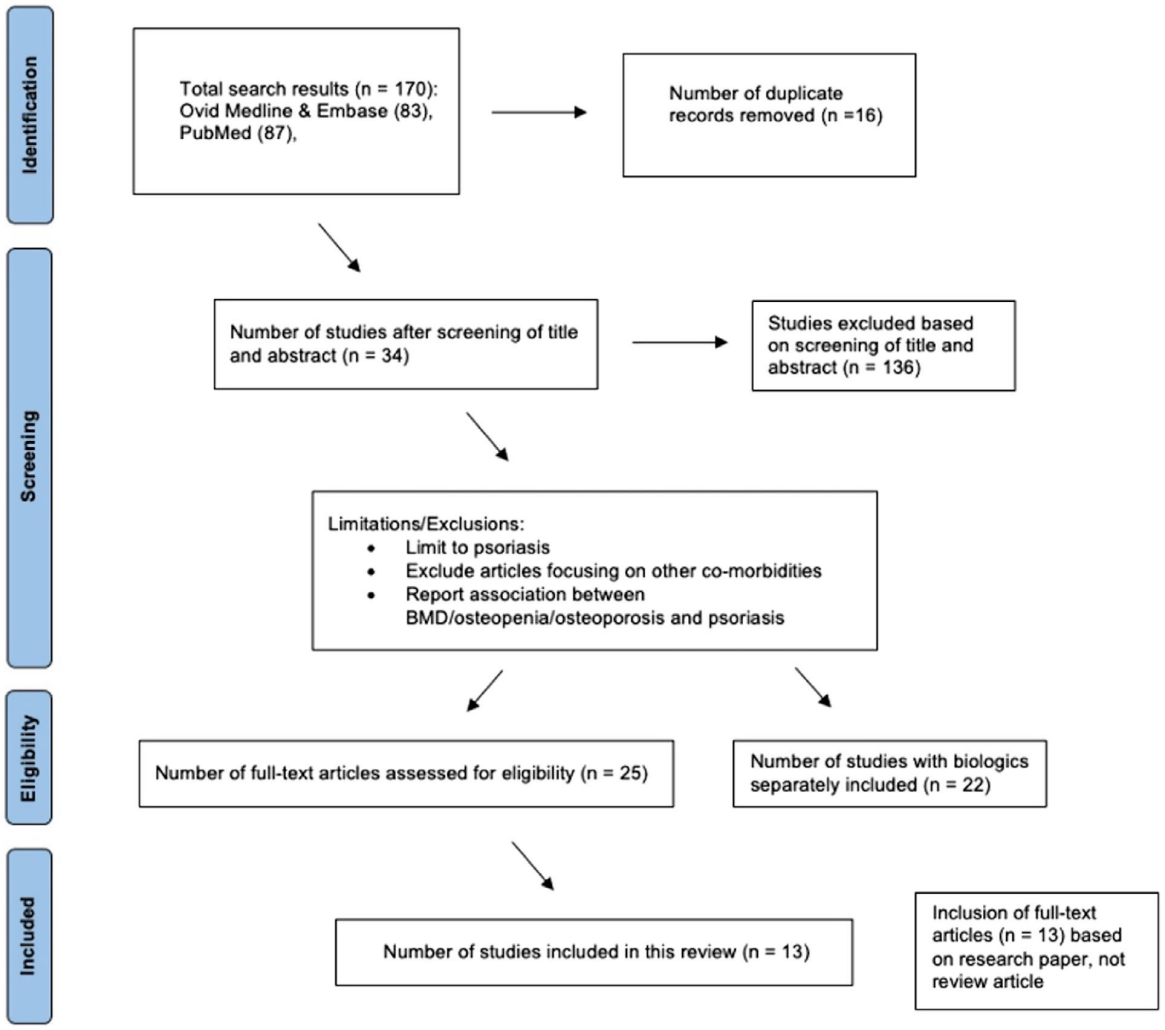
PRISMA flow diagram of literature search strategy. [Colour figure can be viewed at wileyonlinelibrary.com]

A literature search was conducted using Medline, PubMed and Embase databases from all years to date (Fig. 2). Search terms included: ‘prevalence’, ‘psoriasis’, ‘osteopenia’, ‘osteoporosis’ and ‘biologic*.tw’. In total, 13 studies were critically appraised (Table 1).

**Table 1:** Review of the published literature on the prevalence of osteoporosis and osteopenia.

Reference	Type of study	Study period	Country	Patients, *n*	Mean age (years)	M/F, %	Duration of psoriasis (years)	Smoking/alcohol/physical activity	PASI	Treatment for psoriasis	Fractures	Prevalence (%)		Result
												Osteopenia	Osteoporosis	
Dreiher *et al*., 2009^20^	Case–control	1998–2009	Israel	22 771 (controls 14 835)	51–90	52/48	5 ± 3	NA	NA	Acitretin, anti‐TNF‐α, IFN‐α,	NA	NA	12.4 (control 11.2)	Association between psoriasis and osteoporosis was observed in men but not women
Pedreira *et al*., 2011^21^	Cross‐sectional	2011	Brazil	52	61.4 ± 9.1	NA	21.8 ± 17.8	Smoking 21.2%	3.2 ± 3.4	Topical corticosteroids	Fractures 28.8%	NA	NA	Psoriasis and psoriatic arthritis patients have higher risk of fragility fractures and developing metabolic diseases
Balato *et al*., 2012^22^	Prospective cohort	2012	Italy	102	17–54	55/45	NA	NA	NA	NA	NA	24	5	Although clinical data exhibited the association between osteoporosis and psoriasis, there is a lack of abundant clinical evidence in this area
Keller *et al*., 2013^3^	Case–control	2000	Taiwan	79 680 (controls 52 521)	66.1 ± 12.2	23/77	NA	NA	NA	Systemic corticosteroids	NA	NA	1.68	The study found an association between osteoporosis and prior psoriasis among both men and women
D'Epiro *et al*., 2014^16^	Prospective cohort	2014	Italy	43	NA	57/43	13.6	NA	15.9	Ciclosporin A 16%	NA	66	18	Patients with psoriasis are at a higher risk of developing osteopenia/osteoporosis, increasing with duration of psoriasis
Kincse *et al*., 2015^23^	Cross‐sectional	2013	Hungary	72	58.5 ± 11.6	40/32	NA	NA	NA	Biologics ustekinumab, infliximab, adalimumab	NA	NA	32 M, 23 F	The study found an inverse relationship between vitamin D or BMD and psoriasis involvement, stressing the need for routine monitoring and screening
Modalsli *et al*., 2017^24^	Cross‐sectional	2006–2008	Norway	48 194	≥ 20	45/55	NA	Smoking 25%	NA	Topical corticosteroids 14%	NA	NA	13	Large population‐based prospective study found no association between psoriasis and risk of fractures/osteoporosis
Kathuria *et al*., 2017^18^	Cross‐sectional	2006–2012	USA	183 725	54.4 ± 0.1	55/45	NA	NA	NA	NA	Pathological fractures 0.61%; vertebral fractures 0.56%; femoral fractures 1%	0.67	3.3	Both psoriasis and psoriatic arthritis are associated with osteopenia, osteoporosis, osteomalacia and multiple types of fractures
Lajevardi *et al*., 2017^25^	Cross‐sectional	2011–2012	Iran	64	44 ± 17	53/47	27 ± 5	Smoking 20%; physical activity 28%	5.5 ± 4.7	NA	NA	43.8	12.5	Patients with psoriasis had decreased BMD, more significant in men
Martinez‐Lopez *et al*., 2018^26^	Cross‐sectional	2016	Spain	185	48 ± 14	57/43	12 ± 6	NA	7 ± 5	Systemic and biologic treatment	NA	NA	68.9 M, 76.1 F	This cross‐sectional study reported that levels of BMD in patients with psoriasis were situated halfway compared with controls
Freier *et al*., 2018^27^	Prospective cohort	2015	Germany	55	60 ± 12	31/69	16 ± 13	Smoking 59%	NA	Topical corticosteroids 27%	Osteoporotic fractures 33%	38	16	The current literature on the prevalence of osteoporosis in patients with psoriasis necessitate more investigations
Freier *et al*., 2019^28^	Prospective cohort	2015	Germany	103	62 ± 10	33/67	17 ± 13	Regular physical activity 41%; has movement restriction 53%	NA	SEC 18%; MTX 42%; SEC + MTX 8%	Peripheral fragility fractures 34%; vertebral fractures 11%	26	19	The prevalence of osteoporosis in patients with psoriasis or psoriatic arthritis was similar to the normal population
Lee *et al*., 2021^29^	Case–control	2002–2013	South Korea	79 212 (controls 79 212)	≥ 40	49/51	NA	Smoking 13%; alcohol 78% (< 1 time per week), 22% ≥ 1 time per week)	NA	Acitretin, corticosteroids, ciclosporin, MTX	NA	NA	5.1 (control 4.1)	Both case–control studies identified an increased risk of osteoporosis in patients of both sexes with psoriasis

AZA, azathioprine; Cs, ciclosporin; IFN, interferon; MTX, methotrexate; NA, not applicable; PASI, Psoriasis Area and Severity Index; SEC, secukinumab; SCS, systemic corticosteroids; TNF, tumour necrosis factor.

## Prevalence of osteoporosis and osteopenia in patients with psoriasis

The first case–control study was conducted in Israel in 2009 by Dreiher *et al*.,^20^ enrolled 22 771 patients aged 51–90 years with a psoriasis duration of 5 ± 3 years, and 14 835 matched controls. Male patients with psoriasis had a higher prevalence of osteoporosis compared with controls (3.1% vs. 1.7%, OR = 1.86, *P* < 0.001).

In 2011, Pedreira *et al*.^21^ performed a cross‐sectional study of 52 patients in Brazil, and reported a higher incidence of fractures and metabolic diseases in patients with psoriasis and patients with psoriatic arthritis.

Balato *et al*.^22^ conducted a prospective cohort study in 2012 in Italy on 102 patients with psoriasis, and identified an association between osteoporosis and psoriasis, with 5% prevalence.

A population‐based case–control study by Keller *et al*.^3^ in Taiwan enrolled 79 680 patients with psoriasis and 52 521 controls, and found a higher osteoporosis prevalence in patients with psoriasis (1.5% vs. 0.87%, OR = 1.65, *P* < 0.001) compared with controls (*n* = 52 521).

There was a proposal from D'Epiro *et al*.^16^ that the higher risk of developing osteopenia and osteoporosis was linked with increased duration of psoriasis. A 2014 prospective cohort study of 43 patients in Italy by D'Epiro *et al*.^16^ found a longer duration of psoriasis in patients with osteopenia or osteoporosis than in patients with psoriasis with a normal T score (17 years vs. 8.8 years).^16^ Among patients with moderate to severe psoriasis, 66% had osteopenia and 18% has osteoporosis of the lumbar spine (L1–L4) and/or femoral neck.^16^

In 2015, Kincse *et al*.^23^ published a cross‐sectional study from Hungary of 185 patients with a shorter duration of psoriasis (12 ± 6 years) and found a reduced BMD in patients with mild psoriasis compared with matched controls.^23^ This study also recruited patients with moderate to severe psoriasis requiring systemic treatments, and found an inverse relationship between vitamin D and both body mass index and severity of skin involvement.^23^ Nearly two‐thirds (63%) of patients with psoriasis had vitamin D deficiency and BMD loss, and BMD was higher in patients with psoriatic arthritis than in patients with psoriasis alone.^23^

A 2016 Norwegian study by Modalsli *et al*.^24^ found no clear association in 48 194 patients between psoriasis and either BMD T score or osteoporosis prevalence. In 2017, a population‐based cross‐sectional study in the USA by Kathuria *et al*.^18^ assessed 183 725 patients with psoriasis (mean age: 54.4 ± 1 years) and ascertained the prevalence of osteoporosis to be 3.3%. This large sample size contributed to the general association not only between osteoporosis and psoriasis,^3^, ^21^ but also between osteoporosis and other comorbidities such as psoriatic arthritis and fractures.^18^

In 2017, Lajevardi *et al*.^25^ reported 64 patients aged 44 ± 17 years with chronic psoriasis (duration 27 ± 5 years in a cross‐sectional study in Iran, and found that BMD levels were lower in males,^25^ similar to the results from the previous study by Dreiher *et al*.^20^ The prevalence of osteopenia (43.8%) and osteoporosis (12.5%) showed that BMD reduction was associated with psoriasis,^23^, ^26^ smoking and lack of physical activity.^25^

Freier *et al*.^27^, ^28^ conducted two prospective cohort studies in Germany, investigating women aged around 60 with mild to severe psoriasis, over a period of 2 years. The prevalence of osteopenia and osteoporosis in patients with psoriasis was similar to the normal population, which contrast with the existing evidence at the time, and the authors suggest that further investigation was needed.^27^, ^28^ The study included only women and mild cases, potentially skewing the data.

A South Korean case–control study in 2021 by Lee *et al*.^29^ analysed 79 212 control‐matched patients with psoriasis with a mean age of 40 years. Osteoporosis was higher in patients with psoriasis compared with controls (5.1% vs. 4.1%, OR = 1.21) and the increased risk of osteoporosis among patients with psoriasis aged ≥ 40 years was similar in both sexes.^29^

In light of the current literature, there is an increased risk of osteopenia and osteoporosis in patients with extensive psoriasis who have had psoriasis for a long time, compounded by other lifestyle and genetic factors. It suggests that prophylactic measures such as vitamin D supplementation and increasing weight‐bearing exercises can help but patients with extensive psoriasis and prolonged systemic inflammation may require long‐term management.

## Limitations of the study

The review showed that there are inconsistencies in the association of psoriasis and osteoporosis. Small sample sizes^16^, ^21^, ^23^, ^25^, ^27^ affect the reliability and generalizability of the results, and missing patient information can further bias results^3^, ^18^, ^22^, ^23^, ^29^ (Table 1). Lee *et al*.^29^ did not provide details on psoriasis type, severity or duration.^24^, ^26^ Patients with psoriasis are more likely to have concurrent diagnoses such as osteoporosis, but they are also more likely to have increased exposure to medical professionals, thus there is a risk of surveillance bias.^3^

## Conclusion

The limited studies conducted on the association between psoriasis and osteoporosis have not assessed the prevalence of BMD in patients with psoriasis on biologics. Further studies are required to determine how chronic psoriasis may affect BMD and lead to generalized bone loss. This will also help assess the effects of psoriasis on BMD and report the levels of vitamin D in patients on biologics, assisting clinicians to consider whether to incorporate bone health into everyday management of patients with psoriasis.

## Conflict of interest

The authors declare that they have no conflicts of interest.

## Funding

None.

## Ethics statement

Ethics approval and informed consent not applicable.

## Data sharing

Data are available from the corresponding author upon request.

## CPD questions

### Learning objective

To understand what is known of the association between psoriasis and osteoporosis.

### Question 1

Which of the following statements about psoriasis is correct?

(a) Psoriasis is an acute inflammatory skin disease.

(b) Psoriasis is linked with multiple comorbidities, excluding cardiovascular disease.

(c) Well‐circumscribed, erythematous and smooth plaques are all characteristics of untreated psoriasis.

(d) Visible psoriatic plaques can cause discomfort and reduce patient quality of life.

(e) Psoriasis occurs predominantly in developing countries.

### Question 2

Which cytokine is not involved in the pathogenesis of psoriasis and osteoporosis?

(a) Tumour necrosis factor (TNF)‐α.

(b) Interleukin (IL)‐23.

(c) IL‐17.

(d) IL‐8.

(e) IL‐6.

### Question 3

Which of the following statements about osteopenia/osteoporosis is false?

(a) Osteoporosis affects men and women equally worldwide.

(b) A patient with a T score of −1.7 is considered osteopenic according to the World Health Organization criteria.

(c) Osteoporosis affects more men than women worldwide.

(d) Early diagnosis and intervention strategies will assist in the prevention of osteoporosis.

(e) Calcium, vitamin D and weight‐bearing exercises will help in the management of osteoporosis.

### Question 4

Which of the following studies identified a sex‐associated relationship between psoriasis and osteoporosis, with men with psoriasis having a higher rate of osteoporosis than women with psoriasis?

(a) Dreiher *et al*. from Israel and Lajervardi *et al*. from Iran.

(b) Martinez‐Lopez *et al*. from Spain.

(c) Balato *et al*. from Italy.

(d) Pedreira *et al*. from Brazil.

(e) Lee *et al*. from South Korea.

### Question 5

Which of the following are not confounders in the association between psoriasis and osteoporosis?

(a) Patients with psoriasis cover up to avoid embarrassment.

(b) Patients are treated with topical steroids for years.

(c) Patients with severe psoriasis are insulin resistant and overweight with less exercise.

(d) Males have a higher prevalence of osteoporosis than females.

(e) Females have a higher prevalence of osteoporosis than males.

## Instructions for answering questions

This learning activity is freely available online at http://www.wileyhealthlearning.com/ced

Users are encouraged to

- Read the article in print or online, paying particular attention to the learning points and any author conflict of interest disclosures.
- Reflect on the article.
- Register or login online at http://www.wileyhealthlearning.com/ced and answer the CPD questions.
- Complete the required evaluation component of the activity.

Once the test is passed, you will receive a certificate and the learning activity can be added to your RCP CPD diary as a self‐certified entry.

This activity will be available for CPD credit for 2 years following its publication date. At that time, it will be reviewed and potentially updated and extended for an additional period.
